# 2175: An exploratory study of how physicians’ identities inform clinical practice

**DOI:** 10.1017/cts.2017.164

**Published:** 2018-05-10

**Authors:** Candace Chow, Carrie L. Byington, Lenora M. Olson, Karl Ramirez, Shiya Zeng, Ana Maria Lopez

## Abstract

OBJECTIVES/SPECIFIC AIMS: Knowing how to deliver culturally responsive care is of increasing importance as the nation’s patient population diversifies. However, unless cultural competence is taught with an emphasis on self-awareness (Wear, 2007) and critical consciousness (Kumagai and Lypson, 2009) learners find this education ineffective (Beagan, 2003). This study examines how physicians perceive their own social identities (eg, race, socio-economic status, gender, sexual orientation, religion, years of experience) and how these self-perceptions influence physician’s understandings of how to practice culturally responsive care. METHODS/STUDY POPULATION: This exploratory study took place at a university in the Intermountain West. We employed a qualitative case study method to investigate how academic physicians think about their identities and approaches to clinical care and research through interviews and observations. In total, 25 participants were enrolled in our study, with efforts to recruit a diverse sample with respect to gender and race as well as years of experience and specialty. Transcriptions of interviews and observations were coded using grounded theory. One major code that emerged was defining experiences: instances where physicians reflected on both personal and professional life encounters that have influenced how they think about themselves, how they understand an aspect of their identity, or why this identity matters. RESULTS/ANTICIPATED RESULTS: Two main themes emerged from an analysis of the codes that show how physicians think about their identities and their approaches to practice. (1) Physicians with nondominant identities (women, non-White) could more easily explain what these identities mean to them than those with dominant identities (men, White). For example, women in medicine had much to say about being a woman in medicine, but men had barely anything to say about being a man in medicine. (2) There was a positive trend between the number of defining experiences a physician encountered in life and the number of connections they made between their identities and the manner in which they practiced, both clinically and academically. It appeared that physicians who have few defining experiences made few connections between identity and practice, those with a moderate number of experiences made a moderate number of connections, and those with many experiences made many connections. Physicians who mentioned having many defining experiences were more likely to be able to articulate how those experiences were incorporated into their approaches to patient care. DISCUSSION/SIGNIFICANCE OF IMPACT: (1) According to literature in multicultural education, those with dominant identities do not think about their identities because they do not have to (Johnson, 2001). One privilege of being part of the majority is not having to think about life from a minority perspective. This helps to explain why women and non-White physicians in this study had more anecdotes to share about these identities—because they have had defining experiences that prompt reflection on these identities. (2) We propose that struggles and conflict are what compel physicians to reflect on their practice (Eva *et al*., 2012). Our findings suggest that physicians are more prepared to apply what they have learned from their own identity struggles in delivering culturally responsive care when they have had more opportunities to reflect on these identities and situations. Findings from this study have implications for transforming approaches to medical education. We suggest that medical education should provide learners with the opportunity to reflect on their life experience, and that providers may need explicit instruction on how to make connections between their experiences and their practice.

